# Supplementary material for: Health-related quality of life among cervical cancer survivors at a tertiary hospital in Ghana
Source: PLoS One. 2022 Jun 3;17(6):e0268831. doi: 10.1371/journal.pone.0268831 (PMC9165899; doi:10.1371/journal.pone.0268831)
Supplement: Attachment — Submitted filename: 1. marcheditionPLOSrebuttal Letter.docx [file pone.0268831.s003.docx]

**Department of Obstetrics and Gynaecology,**

**Komfo Anokye Teaching Hospital,**

**P.O. Box KS 1934,**

**Kumasi, Ghana.**

Email : [kwabena.amo-antwi@knust.edu.gh](mailto:kwabena.amo-antwi@knust.edu.gh)	[amoantwikwabena@yahoo.com](mailto:amoantwikwabena@yahoo.com)

**March 17, 2022**

**Editor-in-Chief**

**PLOS ONE**

**Dear Sir,**

We are grateful for allowing us to submit a revised manuscript titled ‘‘**Health-related quality of life among cervical cancer survivors at a tertiary hospital in Ghana**.”

We appreciate the time and effort dedicated to providing feedback on our manuscript and the insightful comments and suggestions given to help improve the quality of the manuscript.

We have incorporated the suggestions and answered the queries raised by the reviewers, especially on the methods and materials section. These changes and additional text have been highlighted in blue, and the corresponding lines and page numbers are attached to each query.

Please see below a point-by-point response to the reviewers’ comments or considerations.

Kind regards

**Kwabena Amo-Antwi FWACS FGCS**

Corresponding Author

**Additional Editor comments:**

**Query:** Ethical issue: verbal consent of the participants is a major concern. Do you have any evidence to show that you have gotten the content from the participants?

**Authors response to Editor:** Thank you immensely for your concern. Given that most interviews were conducted over the phone, we discussed this and agreed that the most practical way of obtaining informed consent was the verbal consent. The research protocol including the verbal consent procedures was approved by the Committee on Human Research, Publications and Ethics (CHRPE), Kwame Nkrumah University of Science and Technology and KATH (CHRPE/AP/661/19). A study by Karasik et al (PLoS ONE, 2018: 13(10): e0204428) used a similar approach. We have attached a copy of the patient information sheet and consent form. **Please refer to lines # 266-274, page 12**

**Reviewers' Comments to the Authors:**

**Reviewer 1**

1. **Query:** Recommend the use of Dunn's pairwise comparison to elicit differences in the scores of overall QOL (GHS), financial impact, body image, and symptom experience regarding primary treatment received by the survivors.

**Authors’ response**: Thank you very much for your recommendation. We have included Dunn's pairwise comparison, which further confirmed a difference in the score for overall QOL (p = 0.0036), financial impact (p = 0.0013), body image (p = 0.0013), and symptom experience (p = 0.0068) regarding surgery and chemoradiation (table 5). However, women who had surgery differed from radiation-alone treatment based on body image (p = 0.0212) and financial impact (p = 0.0206). The output for the Dunn's pairwise comparison is provided by table 5. **Please refer to Line #363-368, page 20**

**Reviewer 2**

**Minor considerations**

1. **Query:** The introduction and method sections are not well organized! There is much less-relevant information in the methods and materials section.

**Authors’ response:** The write up on the method and materials section has been revised and made more relevant to the study. Steps have been taken to increase the reproducibility of the methods and materials section.

2. **Query:** Definition of survivors is required. Who are your survivors in your study? Are there both short-term and long-term survivors in your study sample?

**Authors’ response**: Survivorship has now been defined in the methodology. “Cancer survivorship begins at the moment of diagnosis and continues through the cancer trajectories (reference 24). Women who survived cervical cancer of 5 years and beyond after diagnosis, with or without and treatment, were defined as long term survivors. Short-term survivors were those who had not lived for a period not up to five years after cancer diagnosis, with or without treatment”. **Please refer to Line #184 - 187, on page 8**

Defining survivorship necessitated the re-categorisation of the follow-up period and taking cognisance of the cancer diagnosis date (instead of the date of treatment completion). There were short- and long-term cervical cancer survivors. **Please refer to table 1, Line #290 - 294, on page 13**

3. **Query:** It is multivariable logistic regression (not multivariate)

**Authors’ response**: We have replaced “multivariate logistic regression” with “multivariable logistic regression”. **Please refer to Lines #47, #262, #371 and #393 on Pages 2, 11, 20 and 22, respectively.** Thank you.

4. **Query:** I failed to find any definition of COR and AOR in your study. What are COR and AOR?

**Authors’ response**: We have defined and stated how crude odds ratio (COR) and adjusted odds ratio (AOR), symbolized as OR* and OR†, respectively, were obtained in the statistical analysis section of the manuscript. **Please refer to line # 60 on page 3 and Table 3 on page 21**. And again, they were defined below **Table 3**. Thank you.

5. **Query:** Use of the stars in your results is a little bit non relevant. As mostly one star (*) is used to show significance at 0.05 level and two stars (**) at 0.01 level.

**Authors’ response**: We have removed stars used to notate p-values in the results section. **Please refer to table 6, Line #378- 396, on pages 21 and 22.**

**Major considerations**

1. **Query:** This study was presented in a way that your sample size is representative of survivors in Kumasi, Ghana. However, it seems there is a lack of external validity in your study. The sample size of this study is not representative sample of cervical cancer survivors in Ghana, while you covering this in your paper. Thus, you cannot generalize your results.

**Authors’ response**: Thank you very much, we acknowledged this in the limitations of the study: “The findings from this single tertiary institution cannot be generalized to the population of cervical cancer survivors in Kumasi, Ghana or in other LMICs, due to selection bias”. **Please refer to Line #474 - 475, on page 25.**

2. **Query:** On top of that consider that fact that your design is cross-sectional study and prediction is not more accurate in the design, there you need to take more caution when interpreting your results and concluding.

**Authors’ response**: Thank you very much. This has also been acknowledged in the limitations of the study: Cancer survivors' health-related quality of life changes over time; hence, a cross-sectional design is limited in its ability to detect the pattern of overall quality of life across the cancer trajectory. Please **refer to Line #472 - 474, on pages 25.**

3. **Query:** There is an explanation of adequate sample size but not supported well in the text. As a role of thumb, you need more sample size to conduct your study based on the number of predictors and variables that you entered in your model. I checked the study you referred (reference #27) to check the effect size, but that study was not available. It seems that 3.5 difference in scores is relatively low (assuming your effect size value=3.5). Also, there is small number of samples in some categories such as type of treatments.

**Authors’ response**: We assumed that our study's overall QOL score was similar to that observed by Khalil et al. (Quality of life in long-term cervical cancer survivors: results from a single institution, Gynecol Oncol Res Pract. 2015; 2:7.). Sample size calculation was based on the standard deviation of the study mean. Desiring a margin of error of 3.5 percentage points from the actual population overall QoL score, an estimated sample size of 148 had adequate power to detect the sample’s overall QoL score. Using the formula below:

n = (Z^2^. SD^2^) / ME^2^ = (1.97)^2^. (21.7)^2^ /3.5^2^ = (3.8416) (470.89)/12.25=147.67.

[n-sample size, Z=1.96 for 95% confidence interval, ME-margin of error, SD-standard deviation].

However, we do agree that the small number of patients in some categories may limit generalizability of the study findings. We have added this to the limitations of the study. Thank you

4. **Query:** The outcome is HRQoL! Then you need to determine your sample size based on the poor or good qualified (for instance) that you defined it as poor QOL if scores < 0.25 and good score>0.75. then you need to find out the prevalence (using sample size formula based on proportion).

**Authors’ response**: The primary outcome measure is the overall QOL. Please the sample size calculation was based on standard deviation, a similar approach was used by Khalil et al. (2015), an open access article.

5. **Query:** Is the cut point of <0.25 (and >0.75) supported in the literature?

**Authors’ response**: Yes please. Araya et al (Health Qual Life Outcomes. 2020, 16;18(1):72) also dichotomized EORTC QLQ-C30 and QLQ-CX24 domain scores. Please see reference number 28. **Please refer to lines #557-559#, on page 28.**

6. **Query:** Results are repeated in discussion!

**Authors’ response**: The discussion section has been revised, the organization improved and findings that were repeated in the discussion deleted. Thank you.

**Query:** Conclusion is less relevant to your study. Consider the fact that your design is cross sectional. Also, concluding on survival is completely irrelevant.

**Authors’ response**: The conclusion has been revised to reflect the objective of the study.

7. **Query:** Multivariable logistic regression is not clear. What are your predictors here? Are you using outcomes item (QoL items) as predictors (e.g., pain, diarrheoa) and outcome (Global QoL) of your model!

**Authors’ response**: The dependent variable in the model overall QOL (GHS) score, the primary outcome measure. The predictor variables were the other QOL subscales (e.g., pain diarrhoea), sociodemographic, clinical and treatment factors.
